# Hypercaloric low-carbohydrate high-fat diet protects against the development of nonalcoholic fatty liver disease in obese mice in contrast to isocaloric Western diet

**DOI:** 10.3389/fnut.2024.1366883

**Published:** 2024-03-20

**Authors:** Anouk Charlot, Anthony Bringolf, Joris Mallard, Anne-Laure Charles, Nathalie Niederhoffer, Delphine Duteil, Allan F. Pagano, Bernard Geny, Joffrey Zoll

**Affiliations:** ^1^Biomedicine Research Center of Strasbourg (CRBS), UR 3072, “Mitochondrie, Stress oxydant et Plasticité musculaire”, University of Strasbourg, Strasbourg, France; ^2^Faculty of Sport Sciences, University of Strasbourg, Strasbourg, France; ^3^Institute of Cancerology Strasbourg Europe (ICANS), Strasbourg, France; ^4^Faculty of Medicine, University of Strasbourg, Strasbourg, France; ^5^Biomedicine Research Center of Strasbourg (CRBS), UR7296, NeuroCardiovascular Pharmacology and Toxicology Laboratory (LPTNC), University of Strasbourg, Strasbourg, France; ^6^University of Strasbourg, CNRS, Inserm, IGBMC UMR 7104-UMR-S 1258, Illkirch, France; ^7^Service de Physiologie et explorations fonctionnelles, University Hospital of Strasbourg, Strasbourg, France

**Keywords:** obesity, non-alcoholic fatty liver disease, low-carbohydrate diet, Western diet, insulin

## Abstract

**Objective:**

Obesity and metabolic complications, such as type 2 diabetes and nonalcoholic fatty liver disease (NAFLD), are one of the greatest public health challenges of the 21st century. The major role of high sugar and carbohydrate consumption rather than caloric intake in obesity and NAFLD pathophysiology remains a subject of debate. A low-carbohydrate but high-fat diet (LCHFD) has shown promising results in obesity management, but its effects in preventing NAFLD need to be detailed. This study aims to compare the effects of a LCHFD with a high-fat high-sugar obesogenic Western diet (WD) on the progression of obesity, type 2 diabetes, and nonalcoholic fatty liver disease.

**Methods:**

Male C57BL/6J mice were initially fed a WD for 10 weeks. Subsequently, they were either switched to a LCHFD or maintained on the WD for an additional 6 weeks. Hepatic effects of the diet were explored by histological staining and RT-qPCR.

**Results:**

After the initial 10 weeks WD feeding, LCHF diet demonstrated effectiveness in halting weight gain, maintaining a normal glucose tolerance and insulin levels, in comparison to the WD-fed mice, which developed obesity, glucose intolerance, increased insulin levels and induced NAFLD. In the liver, LCHFD mitigated the accumulation of hepatic triglycerides and the increase in Fasn relative gene expression compared to the WD mice. Beneficial effects of the LCHFD occurred despite a similar calorie intake compared to the WD mice.

**Conclusion:**

Our results emphasize the negative impact of a high sugar/carbohydrate and lipid association for obesity progression and NAFLD development. LCHFD has shown beneficial effects for NAFLD management, notably improving weight management, and maintaining a normal glucose tolerance and liver health.

## 1 Introduction

Over the past decades, the prevalence of obesity has substantially increased, reaching 17% of the adult population in France and 42.4% in the United States (1, 2). If several factors have been attributed to the increased obesity rate, the major contributor is diet composition and particularly the Western diet (WD) (3). The WD is characterized by being hypercaloric, rich in saturated fats, refined carbohydrates, and added sugars and salt (4). The consumption of a WD increases the risk of obesity and metabolic comorbidities, such as type 2 diabetes and nonalcoholic fatty liver disease (NAFLD) (5, 6). Currently, type 2 diabetes affects approximately 463 million adults, while the prevalence of NAFLD is estimated to be between 25 and 30% in the world population (7, 8). The increase of NAFLD prevalence is a major challenge of the 21st century, as NAFLD is the contributor to liver mortality and morbidity which has the fastest growth (9).

Health care guidelines promote lifestyle changes for weight loss and comorbidity reduction (10, 11). Therefore, low-calorie diets have become increasingly popular and widespread, even if their long-term weight loss effectiveness are limited due to a low adherence rate (12, 13). The carbohydrate-insulin model of obesity has been proposed to explain the limited effect of caloric restriction on weight loss; it suggests that the adiposity increase is not due to caloric excess but rather to hyperinsulinemia induced by the consumption of high amounts of sugar and refined carbohydrates (i.e., the WD) (14). Thus, the adverse consequences of elevated sugar and carbohydrate consumption within the WD continue to be a subject of debate and are still not fully understood. In this context, studying a low carbohydrate-high-fat diet (LCHFD) represents a good strategy to better understand whether carbohydrates, not an excess of calories, are indeed the trigger for obesity development. Indeed, the LCHFD is a diet in which carbohydrate intake is largely reduced (10% of daily consumption) while overall caloric intake is unchanged and lipid intake is consequently largely increased (by 70 to 80%) (15, 16). In human, the LCHFD diet, popularly known as the ketogenic diet, has been demonstrated to be safe and effective for obesity management and its related complications, by promoting weight loss and improving glucose metabolism despite maintaining a high caloric amount (17–21).

However, the effects of a LCHFD diet in preventing NAFLD remain poorly understood. Pre-clinical studies have demonstrated that the transition from a WD to a hypercaloric LCHFD diet resulted in a modification of the hepatic lipid metabolism genes expression in favor of an increase in those involved in the fatty acid oxidation and a decrease in those involved in fatty acid storage (22–24). Nevertheless, none of these studies directly focused on the LCHFD diet effects on NAFLD markers, including histological explorations.

The objective of this study was to compare the effects of a LCHFD and WD on the progression of obesity, glucose intolerance, and NAFLD development in diet-induced obese mice. We hypothesize that the LCHFD has the potential to impede the progression from obesity to the development of NAFLD suggesting that it is the association of a high sugar and a high calorie amount consumption, and not simply an excess of calories, that is responsible for the worsening of the metabolic complications. We report here that after WD feeding, the LCHFD mice stopped weight gain and prevented glucose intolerance and NAFLD development. These effects seemed to involve a decrease insulin production in comparison to the Western Diet, preventing the liver from *de novo* lipogenesis activation.

## 2 Materials and methods

### 2.1 Animals and study approval

All experiments were approved by our local ethics committee (CREMEAS, agreement numbers: 2018042013495170) and performed following the Guide for the Care and Use of Laboratory Animal Experiments. Thirty-five 8 weeks-old C57BL/6J male mice (ENVIGO, Gannat, France) were maintained at 22 ± 2°C on a 12 h day/night cycle and housed twice in conventional open-top cages enriched with cotton sticks, shredded paper, and wooden chew sticks. Water and food were provided *ad libitum*.

### 2.2 Experimental design and mouse studies

**Figure 1:**
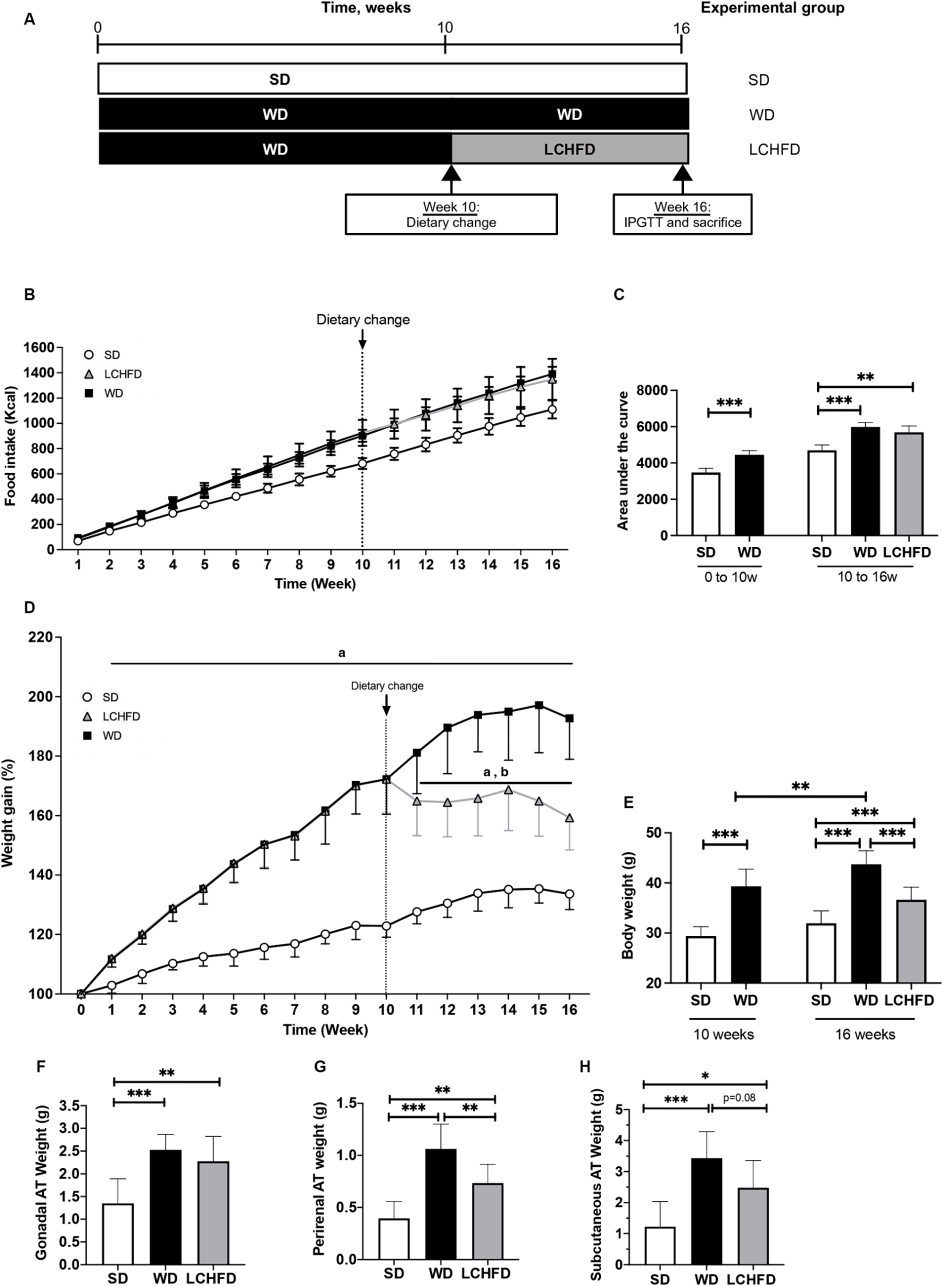
Effect of a WD and LCHFD on caloric intake, weight gain and adipose tissue (AT) accumulation. **(A)** Experimental design of the procedure with the SD group (fed a standard diet throughout the study), WD group (fed a Western diet throughout the study) and LCHFD group (10 weeks of a Western diet feeding followed by 6 weeks of low-carbohydrate high-fat diet feeding). **(B)** Food intake in kcal throughout the procedure. Circles represent the SD group, squares represent the WD group, and triangles represent the LCHFD group. White represents SD feeding, black represents WD feeding, and grey represents LCHFD feeding. **(C)** Quantification of the food intake area under the curve from 0 to 10 weeks and from 0 to 16 weeks. Time refers to weeks. **(D)** Weight gain throughout the procedure expressed in % of the initial body weight. a = different from the SD group, b = different from the WD group. Circles represent the SD group, squares represent the WD group, and triangles represent the LCHFD group. White represents SD feeding, black represents WD feeding, and grey represents LCHFD feeding. **(E)** Body weight of the mice expressed in grams at 10 weeks and 16 weeks. Weight in grams of **(F)** gonadal A. **(G)** perirenal AT and **(H)** Subcutaneous AT. Mean ± SD, *n* = 8–10 * = *p* < 0.05, ** = *p* < 0.01, and *** = *p* < 0.001. SD, standard diet; WD, Western diet; LCHFD, low-carbohydrate high-fat; AT, adipose tissue.

The experimental design is detailed in Figure 1A. Animals were divided into 3 groups: (1) the control group, fed a standard control chow diet (SD) for 16 weeks (SD; 20.5% fat, 15.5% protein and 64% carbohydrates, 3.82 kcal/g, Safe^®^ Diets, *n* = 8); (2) mice fed a high-fat high-sugar WD for 16 weeks (WD 16w; 58.6% fat, 14.4% protein and 27% carbohydrate, 5.52 kcal/g, Safe^®^ Diet, *n* = 9); and (3) LCHFD group, fed a WD for 10 weeks and then fed a LCHFD for 6 weeks (LCHFD; 77% fat, 18.9% protein and 4.2% carbohydrates, 5.55 kcal/g, Safe^®^ Diets, *n* = 10). Body weight and food intake were measured weekly.

Moreover, 9 mice were also fed a WD for 10 weeks and then sacrificed by cervical dislocation, only to establish a baseline for comparison prior to the dietary change (WD 10w). Adipose tissue and liver were harvested and weighted, and the data are available in the Supplementary Figure S1.

Intraperitoneal glucose tolerance tests (IPGTTs) were performed at the 10th week for the WD 10w group and at the 16th week for the SD, WD 16w and LCHFD groups. After 4 h of fasting, glucose (1.5 g/kg iv) was administered and blood glucose concentrations were measured at *t* = 0,15, 30, 45, 60, and 120 min from the tail vein using a glucose meter (AccuChek Performa, Roche, Basel, Switzerland). The area under the curve (AUC) was calculated from the values of IPGTT.

At the end of the 16th week and after 4 h of fasting, the SD, WD and LCHFD mice were placed in a hermetic cage and anesthetized through inhalation of 4% isoflurane (Aerrane, CSP, Cournon, France). Then, mice were euthanized by cervical dislocation and exsanguinated. Blood was collected into a heparinized tube, and plasma was separated by centrifugation (1,000 × g for 10 min at 4°C) and frozen for biochemical analysis. Tissues were harvested, weighed and then either snap-frozen in liquid nitrogen for molecular experiments or cooled in 2-methylbutane immersed in liquid nitrogen for histological staining. All samples were stored at −80°C.

### 2.3 Experimental procedures

#### 2.3.1 Blood sample analyses

Insulin, triglycerides, and total, HDL and LDL cholesterol plasma levels were determined by the Institut Clinique de la Souris (Strasbourg, France) phenotyping platform using an AU-480 automated laboratory workstation (Beckman Coulter France SAS, Villepinte, France). To assess insulin resistance, the HOMA-IR (homeostasis model assessment of insulin resistance) index was calculated as (fasting serum glucose × fasting serum insulin/22.5) (25).

#### 2.3.2 Histological analyses

Liver tissue sections were made at −20°C on a cryostat microtome (Cryostar NX70, Fisher Scientific, Waltham, MA, United States).

For hematoxylin and eosin staining, 10 μm tissue sections were subjected to staining using a standard protocol. First, cryosections were fixed in acetone for 3 s and dried at 37°C for 1 h. Then, they were stained with Harris’ hematoxylin solution for a duration of 2 min, followed by a gentle wash in tap water for 3 min. Next, the sections were bleached in 1% acid alcohol for a brief 2 s period, followed by another wash in tap water for 3 min. Subsequently, counterstaining was performed using an eosin solution for 1 min. Finally, the sections were rinsed in tap water for 3 s, followed by sequential washes in 80% ethanol and 100% ethanol. Finally, the stained sections were mounted with Eukitt medium (Orsatec, Germany). NAS score (NAFLD activity score) was assessed by calculating the score of each NAS component [steatosis (0–3), lobular inflammation (0–3), ballooning (0–2)] (26).

For Oil Red O staining, 10 μm cryosections were initially rehydrated in PBS for 2 min and stained by immersion in Oil Red O solution (Sigma-Aldrich, O0625) for a duration of 3 min. A brief wash in 60% isopropanol for 30 s, followed by a wash in deionized water for 1 min, was applied before counterstaining in Harris’ hematoxylin for 3 min. Then, the cryosections were washed in tap water for 2 min and mounted in Aquatex aqueous medium (Sigma-Aldrich, 108,635). Stained slides were scanned using a Zeiss Apotome.2 microscope (CTK Instruments, Carlsbad, CA, United States), and lipid quantification was assessed using Adobe Photoshop (Adobe Systems, San Jose, CA, United States). The results are expressed as the fold change compared to the SD group.

#### 2.3.3 RNA isolation, reverse transcription, and real-time quantitative PCR

First, total liver RNA was isolated with the Kingfisher Duo Prime (Fisher Scientific, Massachusetts, United States) using the MagMAX^™^ mirVana^™^ Total RNA Isolation Kit according to the manufacturer’s instructions (Applied Biosystems^™^, California, United States) and then stored at −80°C. The quantity and purity of the RNA were assessed with Qubit^™^ RNA Broad Range (BR) and Integrity Quality (IQ) assay kits using the Invitrogen Qubit 4 Fluorometer according to the manufacturer’s instructions (Invitrogen^™^, California, United States). cDNA was synthesized from 2 μg of total liver RNA with Maxima^™^ H Minus cDNA Synthesis Master Mix (Fisher Scientific, Massachusetts, United States). Real-time PCR was performed in triplicate in a total reaction volume of 15 μL using either PowerTrack^™^ SYBR Green Master Mix (Applied Biosystems, California, United States) or in a total reaction volume of 20 μL with Taqman Fast Advanced Master mix (Applied Biosystems, Massachusetts, United States), following the manufacturer’s recommendation. To amplify genes, TaqMan probes were used for the hypoxanthine Phosphoribosyltransferase (Hprt, Mm03024075_m1) and the fatty acid synthase (FASN, Mm00662319_m1), or forward and reverse primers described in Table 1. Real-time PCRs were measured in a QuantStudio 3 Real-Time PCR System (Applied Biosystems^™^, California, United States) using the following cycle parameters: UNG incubation at 50°C for 10 min, enzyme activation at 95°C for 20 s, 40 cycles of denaturation at 95°C for 1 s followed by annealing/extension at 95°C for 20 s. The relative mRNA levels were normalized to Hprt housekeeping gene levels, which were unaffected by the experiment. We used the ΔΔCt method to normalize cycle threshold values for each gene of interest (27).

**Table 1:** Primers for liver qPCR.

Gene	Forward primer (5′–3′)	Reverse primer (3′–5′)
Hprt	GTTGGATACAGGCCAGACTTTGTTG	GATTCAACTTGCGCTCATCTTAGGC
Acadl	GAAGATGTCCGATTGCCAGC	AGTTTATGCTGCACCGTCTGT
Acadm	ATGACAAAAGCGGGGAGTACC	CCATACGCCAACTCTTCGGT
Acc1	AGGCGGATATCTGCTGAGAC	CCAGACATGCTGGATCTCAT
Echs1	GCAAAGCAGGCAGGTCTTGT	TAGCTGCCAGTTCTCAGTGG
Hadh	TCGTGAACCGACTCTTGGTG	ATTTCATGCCACCCGTCCAA
Pparα	ACTACGGAGTTCACGCATGTG	TTGTCGTACACCAGCTTCAGC
Scd1	ATCGCCCCTACGACAAGAAC	AACTCAGAAGCCCAAAGCTCA
Srebp1	GGAACTTTTCCTTAACGTGGGC	ATGAGCTGGAGCATGTCTTCG

Hypoxanthine phosphoribosyltransferase (Hprt), acyl-CoA dehydrogenase long chain (Acadl), acyl-CoA dehydrogenase medium chain (Acadm), acetyl-coA carboxylase 1 (Acc1), enoyl coenzyme A hydratase (Echs1), hydroxyacyl-coenzyme A dehydrogenase (Hadh), peroxisome proliferator activated receptor alpha (Pparα), stearoyl-coA desaturase-1 (Scd1) and sterol regulatory element-binding protein 1 (Srebp1).

### 2.4 Statistical analysis

All data are shown as the mean ± SD. Normal distribution of data was checked using the Shapiro–Wilk test and outliers were checked using ROUT method. Two-group comparisons were assessed with student’s *t*-test, 3-group or more comparisons were made with one-way ANOVA or Kruskal–Wallis tests, followed by Tukey’s or Dunns’ post hoc test. Correlations were assessed with Pearson or Spearman tests, depending on the result of the normality test. All statistical analyses were performed using GraphPad 8^®^ (GraphPad Software, Inc.). Statistical significance is shown as ^*^p < 0.05, ^**^p < 0.01, and ^***^p < 0.001.

## 3 Results

### 3.1 A low-carb high-fat diet stopped weight gain and adipose tissue accumulation following Western diet feeding

Eight-weeks-old C57Bl6/J male mice were fed a WD for 10 weeks. Compared to the SD mice, the WD mice presented a significant increase in caloric intake (Figure 1B), displayed by the area under the curve (*p* < 0.001, Figure 1C), and a statistically significant increase in weight gain (*p* < 0.001, Figure 1D). At the end of the 10th week, the WD mice reached a body weight of 39.3 ± 3.4 g, while the SD mice weighed 29.4 ± 1.8 g (*p* < 0.001, Figure 1E). This significant weight gain was associated with gonadal (+42%, *p* < 0.05) and perirenal AT accumulation (+75%, *p* < 0.01, Supplementary Figure S1), compared to the SD mice. After 10 weeks, the WD mice were separated into two groups, one of which continued with the WD and the other of which transitioned to the LCHFD, for 6 weeks. The WD and LCHFD mice continued to consume a higher caloric amount than the SD mice (*p* < 0.001, Figures 1B,C), without a significant difference between the WD and LCHFD groups.

The WD group exhibited continuous weight gain throughout the study, reaching a significant weight gain of +93% at 16 weeks (*p* < 0.001) compared to the SD group (Figure 1D), and a final body weight of 43.7 g ± 1.0, compared to their 10 weeks weight (*p* < 0.01, Figure 1E). This weight gain was accompanied by a significant increase in gonadal adipose tissue, perirenal adipose tissue and subcutaneous adipose tissue (*p* < 0.001, Figures 1F–H) in comparison with the SD group.

Interestingly, when the mice switched to the LCHFD, weight gain stopped (Figures 1D,E). Indeed, after 6 weeks of the LCHFD, the final body weight of the LCHFD mice was significantly lower than that of the WD mice (*p* < 0.001) and similar to those measured at 10 weeks (Figure 1E). LCHFD mice presented a significantly higher gonadal, perirenal (*p* < 0.001, Figures 1F,G) and subcutaneous AT weight (*p* < 0.05, Figure 1H), compared to the SD mice. Interestingly, the LCHFD mice presented a significantly lower perirenal AT weight (−27%, *p* < 0.01) and a tendency toward a lower subcutaneous AT weight (−22%, *p* = 0.080) in comparison with the WD 16w mice.

### 3.2 A low-carb high-fat diet prevents ongoing Western diet-induced glucose intolerance and dyslipidemia

**Figure 2:**
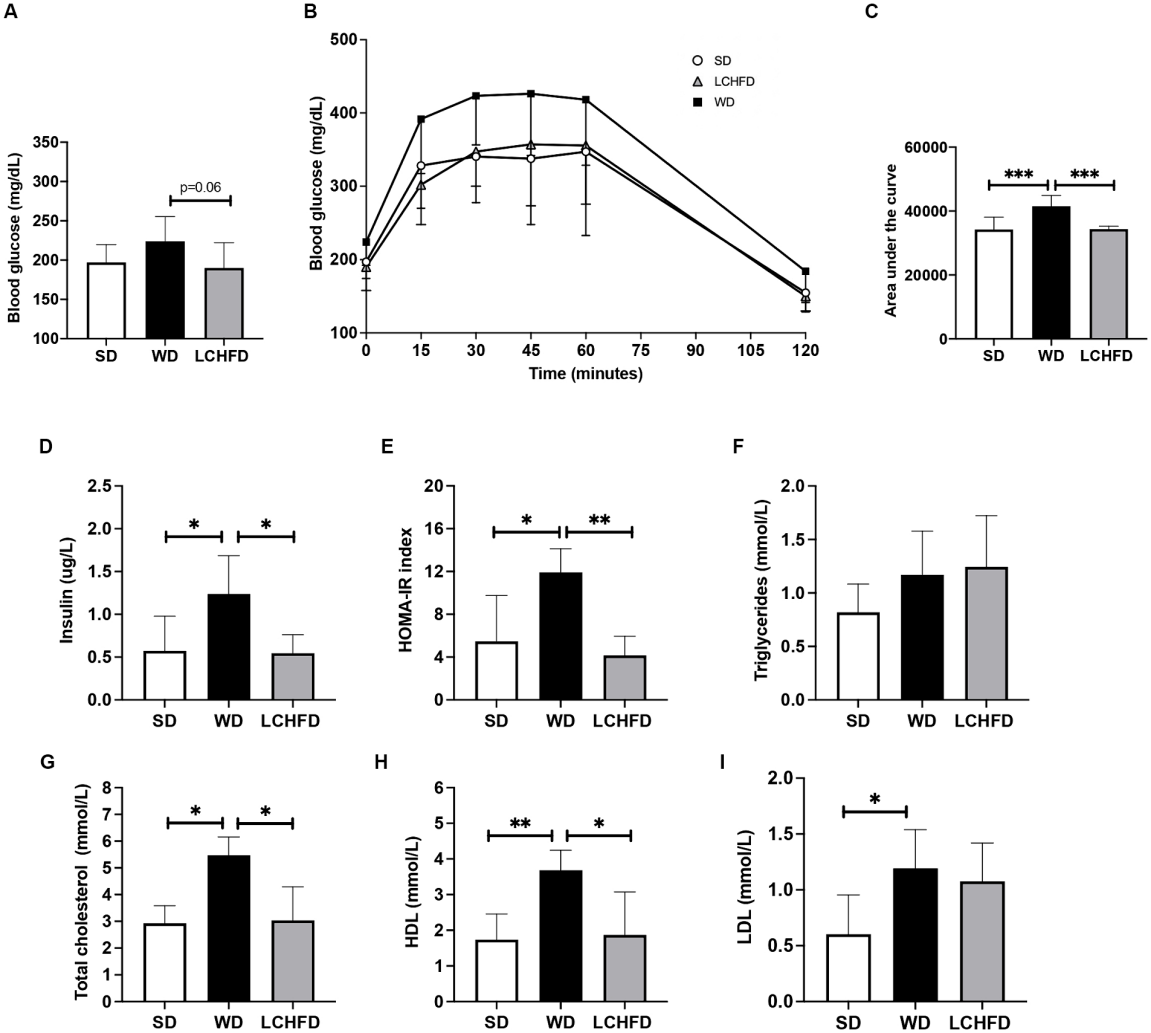
Effects of a WD and LCHFD on glucose tolerance, insulin levels and lipid profiles. **(A)** IPGTTs were performed after 10 weeks of diet (WD 10w) and after 16 weeks (SD, WD 16w and LCHFD groups, *n* = 8–10). **(B)** IPGTT corresponding area under the curve. **(C)** Fasting blood glucose measured from the vein tail after 4 h of fasting (*n* = 8–10). Plasma level measurement of **(D)** fasting insulin. **(E)** Triglycerides. **(F)** Total cholesterol. **(G)** High-density lipoprotein (HDL) cholesterol. **(H)** Low-density lipoprotein (LDL) cholesterol. For blood samples, *n* = 5–6. Mean ± SD * = *p* < 0.05, ** = *p* < 0.01, and *** = *p* < 0.001. SD, standard diet; WD, Western diet, LCHFD, low-carbohydrate high-fat.

Despite exhibiting a similar fasting blood glucose level to those of SD mice (Figure 2A), WD mice exhibited exacerbated abnormal glucose tolerance, the area under the curve quantification confirmed that the WD group had consistently higher glycemia throughout the test, compared to the SD group (*p* < 0.001, Figure 2C). Moreover, insulin levels and the HOMA-IR index of the WD mice were significantly increased compared to those of the SD mice (respectively +143%, *p* < 0.05 and + 118%, *p* < 0.01, Figures 2D,E)

In contrast, the fasting blood glucose levels of the LCHFD mice were tended to be lower than those of the WD mice (−24%, *p* < 0.0595, Figure 2A). The glucose tolerance curve of the LCHFD mice was similar to that of the SD mice (Figure 2B), and the area under the curve was significantly different from that of the WD mice (*p* < 0.001, Figure 2C). Moreover, the insulin levels and the HOMA-IR index of the LCHFD mice remained similar to those of the SD mice and were, respectively, 56 and 65% lower than those of the WD 16w mice (*p* < 0.05, Figures 2D,E).

Regarding lipid profile measurements, 16 weeks of a WD did not impact triglycerides levels (Figure 2F) but induced a substantial increase in total cholesterol (*p* < 0.05), HDL (*p* < 0.01), and LDL (*p* < 0.05) levels in comparison with those of the SD mice (Figures 2G–I). Interestingly, mice who switched to a LCHFD showed a decrease of total cholesterol and HDL levels in comparison with the WD mice (*p* < 0.05), and displayed triglycerides, total cholesterol, LDL HDL levels similar to those of the SD mice (Figures 2G–I).

### 3.3 A low-carb high-fat diet prevents ongoing Western diet-induced hepatic steatosis and *de novo* lipogenesis

As NAFLD is a prevalent comorbidity associated with obesity, we conducted liver analyses. First, we characterized the hepatic effects of 10 weeks of WD feeding. The liver weight of the WD 10w mice was similar to that of the SD mice and histological staining revealed few steatosis spots, without significant hepatic lipid accumulation compared to the SD mice (Supplementary Figure S1).

**Figure 3:**
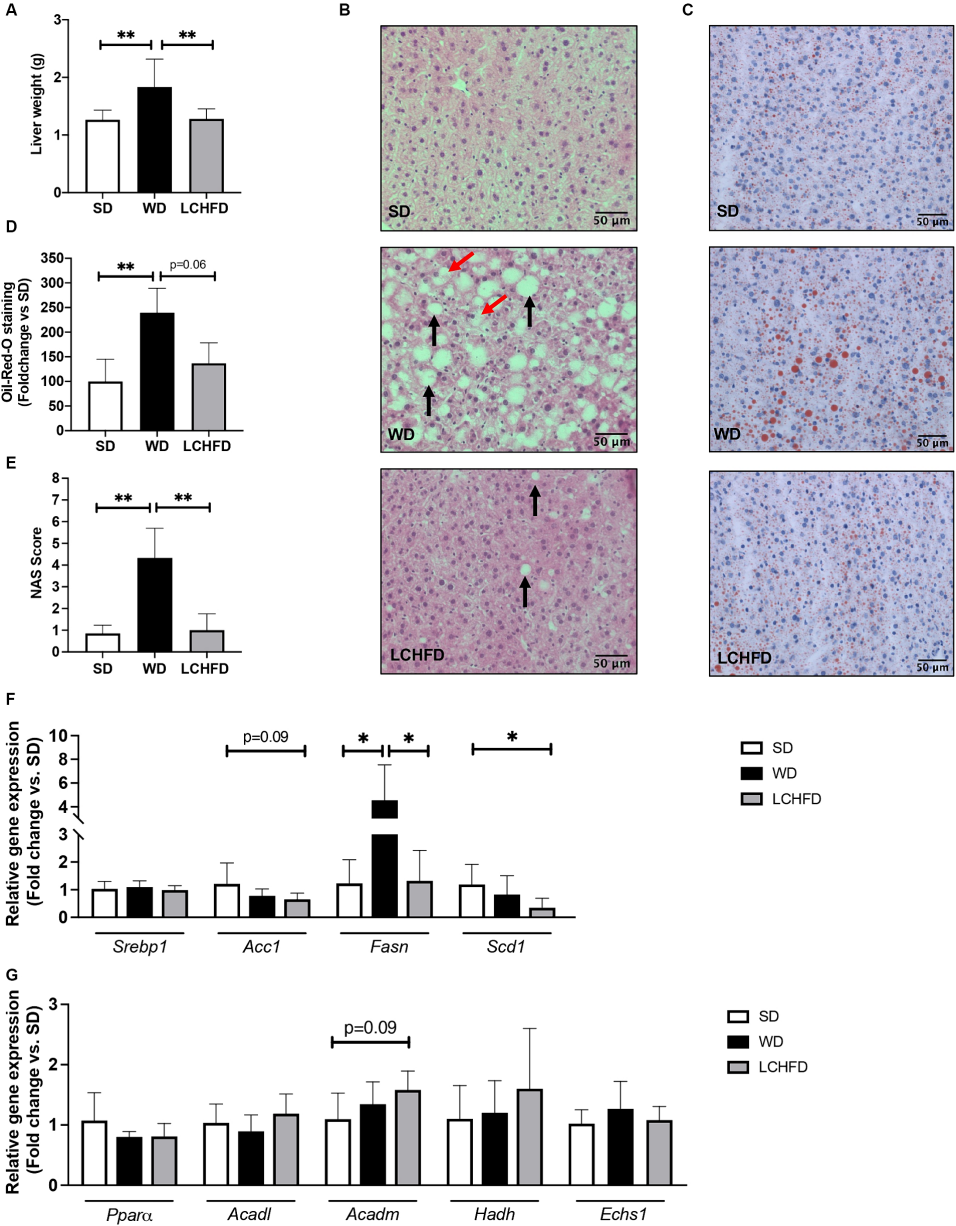
Effects of a WD and LCHFD on liver histology and fatty acid metabolism actor’s gene expression. **(A)** Liver weight in grams, *n* = 8–10. **(B)** Hematoxylin-eosin staining of liver sections. The black arrow indicates steatosis, and the red arrow indicates hepatocyte balloonization. **(C)** Oil Red O staining of liver sections. Lipids are stained red. **(D)** Oil Red O staining quantification, *n* = 4–5. **(E)** NAFLD activity score (NAS) score, *n* = 6–8. Relative gene expression of **(F)** fatty acid oxidation markers including peroxisome proliferator activated receptor alpha (Pparα), acyl-CoA dehydrogenase long chain (Acadl), acyl-CoA dehydrogenase medium chain (Acadm), hydroxyacyl-coenzyme A dehydrogenase (Hadh) and (H) enoyl coenzyme A hydratase (Echs1). Relative gene expression of markers of fatty acid storage **(G)** including sterol regulatory element-binding protein 1 (Srebp1), fatty acid synthase (Fasn) and stearoyl-coA desaturase-1 (Scd1), *n* = 6–8. Mean ± SD * = *p* < 0.05 and ** = *p* < 0.01. SD, standard diet; WD, Western diet; LCHFD, low-carbohydrate high-fat diet.

After 16 weeks, the WD mice developed hepatomegaly with a considerable rise in liver weight compared to the SD (+45%, *p* < 0.01, Figure 3A). Hematoxylin-eosin staining and NAS score revealed NAFLD development, characterized by steatosis and hepatocyte ballooning (*p* < 0.01, Figures 3B,E), along with a significant red lipid droplet accumulation compared to the SD mice (+139%, *p* < 0.05, Figures 3C,D). Interestingly, the LCHFD prevented hepatomegaly, as liver weight in the LCHFD group was comparable to that of the SD group but lower than that of the WD 16w group (Figure 3A, *p* < 0.01). Hepatic histological staining of LCHFD group presented fewer steatosis spots and lower red lipid droplet accumulation compared to WD 16w liver (−100%, *p* < 0.0629, Figures 3B–D). NAS score of LCHFD mice was similar to that of SD mice and lower than the WD group (*p* < 0.01, Figure 3E).

**Figure 4:**
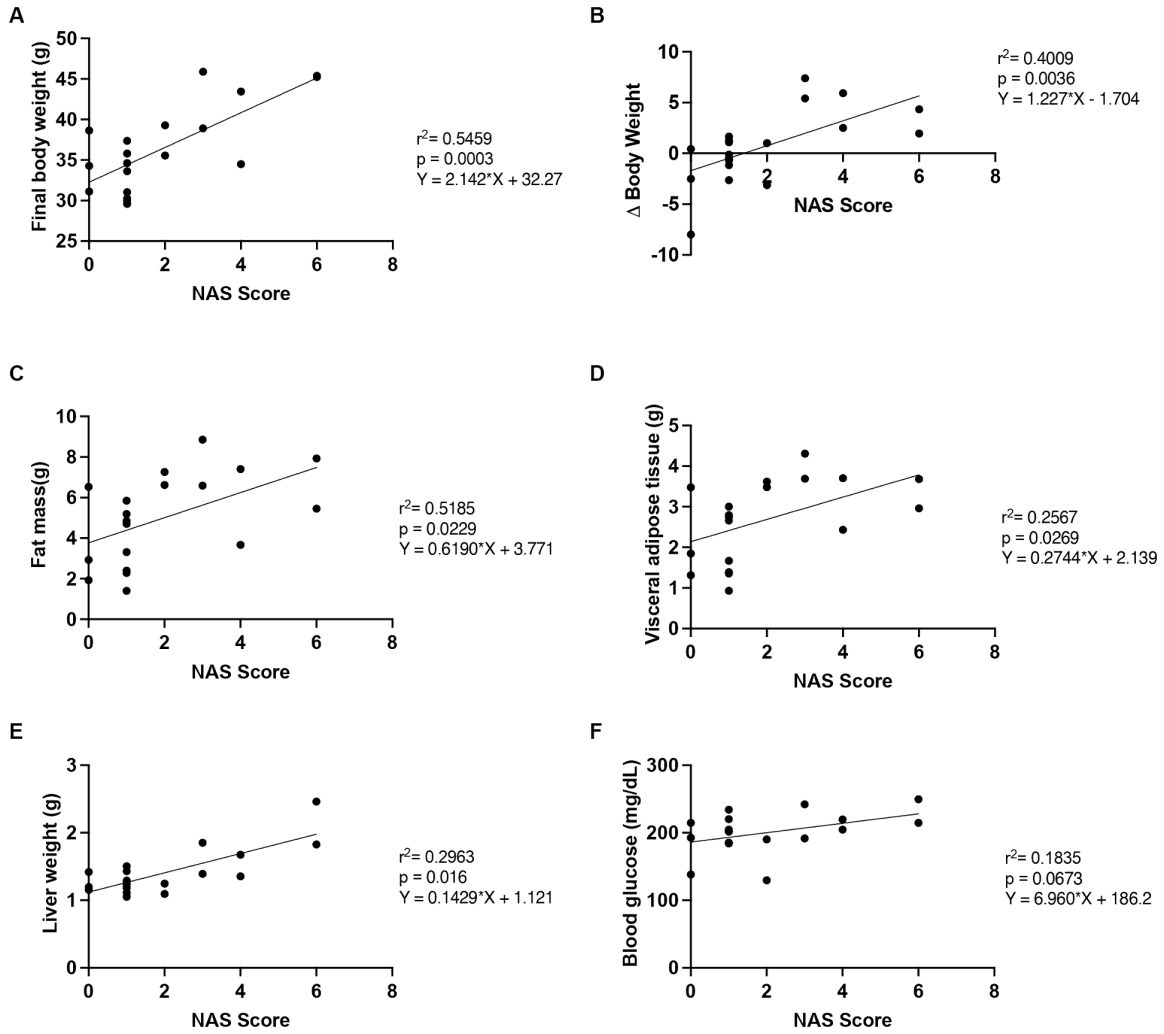
Correlation of NAS score with outcome measures. The relationship between NAS score and **(A)** final body weight, **(B)** Δ body weight (between the 10th and 16th weeks), **(C)** fat mass, **(D)** visceral adipose tissue mass, **(E)** liver weight and **(F)** fasting blood glucose, *n* = 19. SD, standard diet; WD, Western diet; LCHFD, low-carbohydrate high-fat diet.

Regarding the hepatic gene expression of the actors involved in fatty acid metabolism, Fasn gene expression of the WD mice was fourfold higher than that of SD mice (*p* < 0.05) but the relative gene expression of Pparα, Acadl, Acadm, Echs1 or Hadh remains similar to the SD mice (Figures 4E,F). Interestingly, the transition to a LCHFD induced a significant decrease in Fasn gene expression in comparison with the WD mice (*p* < 0.05) and a decrease in Scd1 gene expression in comparison with SD mice (*p* < 0.05). Moreover, mice showed a tendency to decrease Acc1 (*p* = 0.0866) and increase Acadm (*p* = 0.0965) gene expression compared to the SD group (Figures 3F,G).

Finally, we measured the relationship between the NAS score and several markers of progression and improvement of the NAFLD (Figure 4) (28–30).

We found that the final body weight (*r*^2^ = 0.5459, *p* = 0.0003), the Δ body weight (*r*^2^ = 0.4009, *p* = 0.0036), the fat mass (*r*^2^ = 0.2689; *p* = 0.0229), the visceral adipose tissue mass (*r*^2^ = 0.2567; *p* = 0.0269) and the liver weight (*r*^2^ = 0.2963; *p* = 0.016), were correlated with the NAS score. Moreover, the fasting blood glucose tended to correlate with the NAS score (*r*^2^ = 0.1835, *p* = 0.0673).

## 4 Discussion

The effect of dietary macronutrient composition rather than caloric amount on obesity and NAFLD pathophysiology remains a topic of much debate. In this study, we conducted a comparative analysis to assess the metabolic impact of high sugar/carbohydrate amounts in high-calorie diets by studying the effects of hypercaloric WD and LCHFD on obesity progression and complications development. We reported that the WD induced obesity, glucose intolerance, and NAFLD development, whereas a LCHFD limited weight gain, and maintained a normal glucose regulation, insulin levels and hepatic health, preventing the development of all symptoms. These findings suggest that the detrimental effects of the WD are primarily attributed to the combination of sugar and lipids rather than the hypercaloric nature of the diet.

After 10 weeks of a WD, mice displayed a significant increase in weight gain indicating obesity development, but without hepatic complications. These results are consistent with other studies using diet-induced obesity (22–24). When the WD was continued for an additional 6 weeks, mice showed a progression of obesity, associated with several obesity-related disorders, such as hyperinsulinemia, glucose intolerance, dyslipidemia and NAFLD development. In contrast, switching the high-fat high-sugar WD to a low-carbohydrate high-fat diet mitigated WD-induced weight gain, and prevented the development of dyslipidemia, glucose intolerance and NAFLD in mice. Indeed, the LCHFD mice stopped their weight gain, even though their caloric intake was comparable to that of the WD mice and substantially higher than that of the SD mice. The same observations were also reported previously, in diet-induced obesity models when mice were fed *ad libitum* with a LCHFD (22–24). Moreover, a study demonstrated that healthy mice maintaining a hypercaloric LCHFD showed a decrease in weight compared to the SD mice, although LCHFD mice consumed significantly more calories than their SD counterparts (31). Altogether, our results and those of the literature indicate that excess calories alone cannot explain obesity development, although energy imbalance is conventionally stated to be the main driver of weight gain (32). Therefore, macronutrient repartition seems to be a trigger for obesity development, particularly when carbohydrates and sugars are consumed conjointly with lipids.

Interestingly, we found a significant increase of the HDL levels in the WD, compared with both SD and KD mice, while increased HDL levels are commonly associated with a reduction of the cardiovascular risks (33). The increased HDL cholesterol levels have been previously described in C57Bl/6 mice as well as human upon WD feeding (34, 35). This phenomenon may be an adaptive response to the greater need to transport the high lipids content, suggesting that the association between HDL levels and metabolic risk is more complicated than originally proposed, and as such, increased HDL-C levels should be interpreted with care (34). Other studies suggest that LDL and total cholesterol levels should be the gold standard metabolic risk markers for cardiovascular disease, instead of using plasma HDL levels as an indicator of cardiovascular health (36, 37). Therefore, the significant increase of HDL and total cholesterol levels in the WD mice indicates an increase of cardiometabolic risk while LCHFD prevented the development of hypercholesterolemia.

The deleterious effect of carbohydrates has been described in the carbohydrate-insulin model of obesity development, which postulates that the accumulation of adipose tissue is linked to the high secretion of insulin that occurs in response to the consumption of a high-carbohydrate diet (14, 38). Indeed, insulin is an anabolic hormone that promotes the storage of fatty acids in adipose tissue as well as in the liver (39). In our model, the WD mice, which had a significant increase in weight gain, showed hyperinsulinemia and glucose intolerance, while the LCHFD mice stopped the weight gain, had normal glucose regulation, and exhibited fasting insulin values similar to control mice, suggesting that the insulin rise could be the main driver of fat mass accumulation. Our observations are consistent with previous studies on diet-induced obese mice, where the authors showed that a LCHFD promoted fat mass decrease, glucose regulation improvement and insulin decrease (22–24). Moreover, a study conducted on 218 diabetic and overweight patients showed a beneficial effect of a hypercaloric LCHFD on weight loss and glucose regulation, including a significant decrease in insulin levels (18). Therefore, our results and the literature suggest that the beneficial effects of a LCHFD on weight gain are directly due to the decrease in insulin secretion, in response to carbohydrate restriction.

In the liver, the WD and LCHFD mice exhibited opposite metabolic adaptations. Indeed, the WD mice showed a massive accumulation of triglycerides while the LCHFD mice showed a healthy liver, without excessive triglyceride accumulation, steatosis, or ballooning. Interestingly, the correlation study revealed that the development of NAFLD is associated with the body weight, adiposity and liver weight. Weight gain and visceral obesity are important risk factors for the onset of NAFLD (40), so the reduction of weight gain and visceral fat accumulation in LCHFD mice is involved in the protective effect of the LCHFD on NAFLD development. At molecular levels, the two diets also exhibited opposite regulation. WD mice showed a significant increase in Fasn gene expression, which plays a major role in *de novo* lipogenesis, and consequently to NAFLD (41). Conversely, the LCHFD mice showed a hepatic Fasn gene expression similar to the SD mice, a decrease of Scd1 and a tendency to decrease Acc1 gene expression. Interestingly, the control of *de novo* lipogenesis is primarily transcriptional because insulin activates the endoplasmic reticulum membrane-bound transcription factor sterol regulatory element binding protein 1 (SREBP1), which translocates to the nucleus and upregulates the genes involved in the fatty acid biosynthetic pathway and triglycerides synthesis pathway, including Acc1, Fasn and Scd1 (42–44). Although we did not find changes in SREBP1 expression, the modification of its downstream targets suggests the involvement of its pathway in the response to the dietary changes. We hypothesized that the opposite *de novo* lipogenesis gene expression in both the WD and LCHFD mice may be a consequence of a distinct hormonal response due to the macronutrient composition of the diet. The significant increase of insulin induced by the WD could lead to an upregulation of Fasn gene expression and thus, to the increase of hepatic lipid accumulation, while maintaining normal insulin levels does not promote the activation of *de novo* lipogenesis and triglyceride synthesis, protecting the liver from NAFLD development (45, 46). We also focus on the β-oxidation, but the diets did not seem to influence the actors involved in this pathway. However, PPAR⍺ is a transcription factor normally activated by fatty acids, inducing the transcription of a certain number of target genes, including beta-oxidation enzymes (47). It would seem that this pathway is not involved in the opposite metabolic effects observed between the two diets. Therefore, the beneficial effects of LCHFD on obesity and NAFLD seem to be explained by the reduction in insulin secretion, which would limit both weight gain due to the accumulation of lipids in adipose tissue and *de novo* lipogenesis activation, but they not involve an increase of fatty oxidation pathway.

However, our study has some limitations. Firstly, our study design was exclusively intended to investigate the effects of macronutrient changes on insulin regulation and the development of NAFLD. However, the dysregulation of visceral adipose tissue is also involved in the development of obesity-related complications (48). Given the favorable impact of LCHFD on weight gain, there is a compelling interest in extending this study to characterize its effects on visceral adipose tissue, particularly at the molecular level. Furthermore, we did not explore the influence of dietary changes on total energy expenditure, despite it can affect weight gain. Future studies should focus on investigating the impact of LCHFD on metabolic adaptations, using the approach of metabolic cage, for example. Finally, like the other studies that focus on the metabolic effects of LCHFD on obesity, our research only involved male subjects. Considering the hormonal differences between males and females, as well as the sexual dimorphism in response to a WD, future studies should incorporate both sexes to comprehensively determine the effects of LCHFD, including its impact on females.

**Figure 5:**
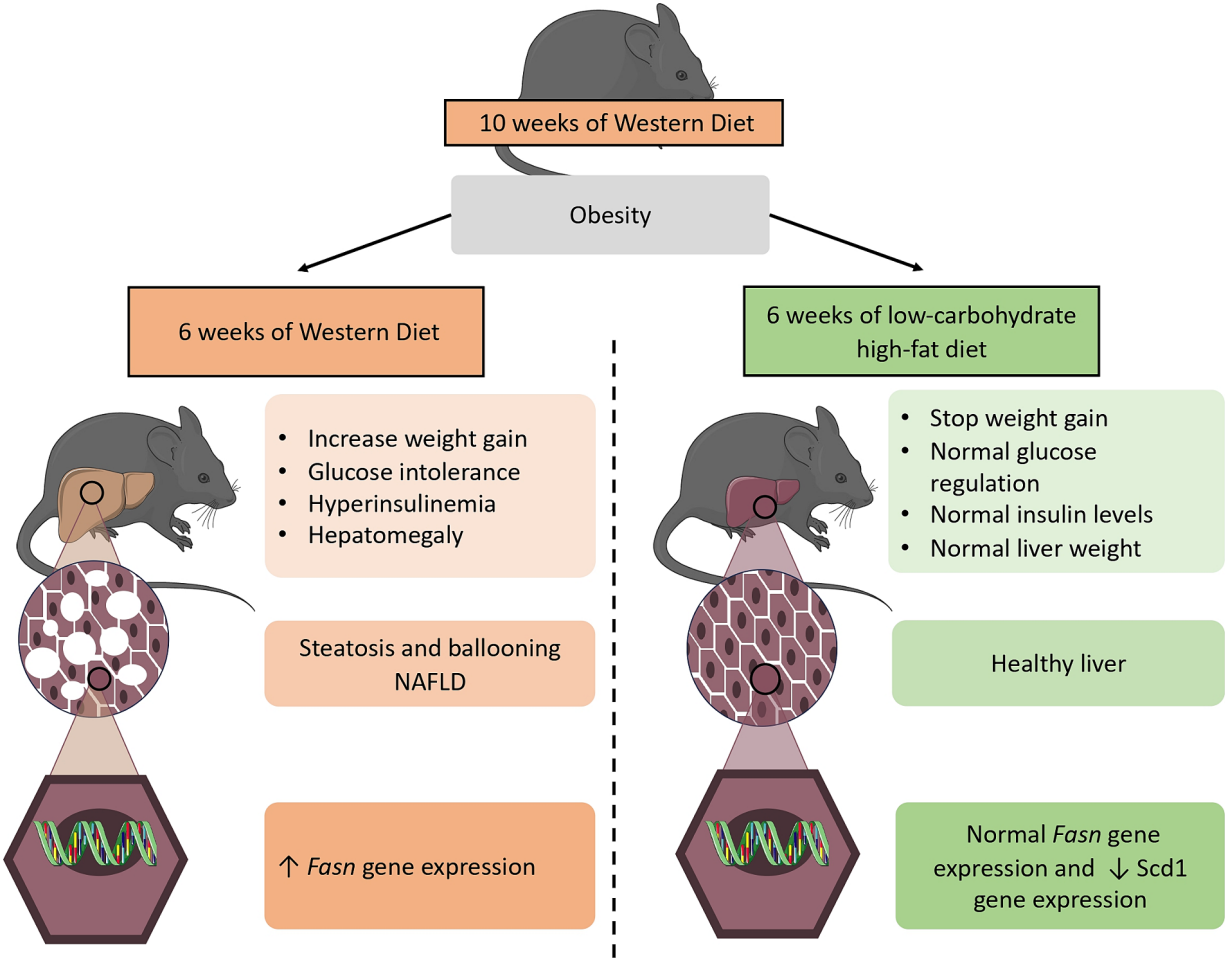
Diagram showing the 6 weeks dietary effects of both a WD and LCHFD on the diet-induced obesity mouse model. The 10 weeks consumption of a high-fat high-sugar WD is responsible for obesity development. When the WD is maintained for 6 additional weeks, weight gain continues to rise, insulin levels are elevated, and mice develop NAFLD, which is associated with an increase in *de novo* lipogenesis Fasn gene expression in the liver. In contrast, a low-carbohydrate high-fat LCHFD stop weight gain and protect from glucose intolerance and NAFLD development. The figure was partly generated using Servier Medical Art (Servier, Creative Commons Attribution 3.0 unported license).

In this study, we therefore demonstrated that two diets with the same caloric amount, but a different macronutrient composition exhibited opposite metabolic responses. On the one hand, the obesogenic WD induces hyperinsulinemia, resulting in an increase in *de novo* lipogenesis activation in the liver. On the other hand, the LCHFD maintains insulin levels and *de novo* lipogenesis Fasn gene expression similar to the control animals, protecting the liver against NAFLD development (Figure 5). Regarding the clinical implication of our study, we emphasize the detrimental effects of high sugar/carbohydrate intake in high-calorie diets and propose that nutritional management be reconsidered to enhance obesity management in patients. Indeed, considering the major impact of high sugar levels in any diet rather than aiming to simply reduce caloric intake may help to understand the following consequences on insulin levels and, ultimately, weight changes and hepatic complications in humans. We demonstrated the beneficial effects of the low-carbohydrate high-fat diet on obesity and NAFLD development, and we believe that healthcare professionals should consider this approach to reduce weight gain and prevent the onset of obesity-related complications such as glucose intolerance and NAFLD.

## Data availability statement

The raw data supporting the conclusions of this article will be made available by the authors, without undue reservation.

## Ethics statement

The animal study was approved by Comité régional d’éthique en matière d’expérimentation animale de Strasbourg (CREMEAS), Strasbourg, France. The study was conducted in accordance with the local legislation and institutional requirements.

## Author contributions

AC: Conceptualization, Investigation, Methodology, Writing – original draft, Writing – review & editing. AB: Investigation, Writing – review & editing. JM: Investigation, Writing – review & editing. A-LC: Investigation, Methodology, Writing – review & editing. NN: Investigation, Writing – review & editing. DD: Investigation, Methodology, Writing – review & editing. AP: Investigation, Methodology, Writing – review & editing. BG: Validation, Writing – review & editing. JZ: Conceptualization, Methodology, Supervision, Writing – original draft, Writing – review & editing.

## Supplementary material

### Supplementary figure 1

Weight of **(A)** gonadal AT, **(B)** perirenal AT, **(C)** subcutaneous AT and **(D)** liver. *n*=8-9. **(E)** Hematoxylin-eosin staining of liver sections. The black arrow indicates steatosis. **(F)** Oil Red O staining quantification. *n*=4-5. **(G)** Oil Red O staining of liver sections. Lipids are stained red. Mean ± SD *=*p*<0.05 and **=*p*<0.01. SD: standard diet group; WD 10w: animal fed a western diet for 10 weeks; AT: adipose tissue.
