# Supplementary figures and images for: Hypercaloric low-carbohydrate high-fat diet protects against the development of nonalcoholic fatty liver disease in obese mice in contrast to isocaloric Western diet
Source: Front Nutr. 2024 Mar 20;11:1366883. doi: 10.3389/fnut.2024.1366883 (PMC10987868; doi:10.3389/fnut.2024.1366883)

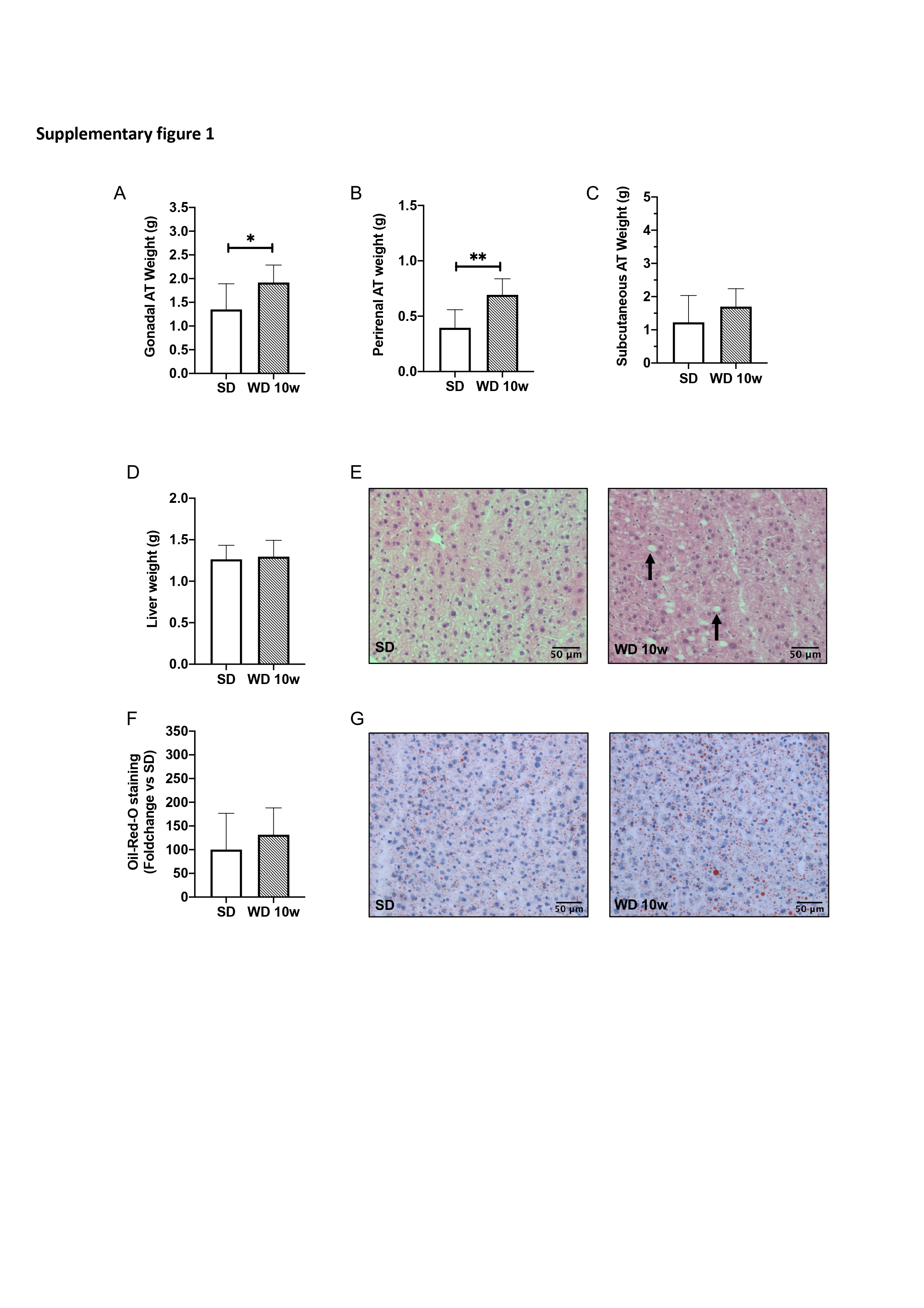

Supplement: Supplementary file 1 [file Image_1.jpeg]
